# Estimating the True Accuracy of Diagnostic Tests for Dengue Infection Using Bayesian Latent Class Models

**DOI:** 10.1371/journal.pone.0050765

**Published:** 2013-01-18

**Authors:** Wirichada Pan-ngum, Stuart D. Blacksell, Yoel Lubell, Sasithon Pukrittayakamee, Mark S. Bailey, H. Janaka de Silva, David G. Lalloo, Nicholas P. J. Day, Lisa J. White, Direk Limmathurotsakul

**Affiliations:** 1 Department of Tropical Hygiene, Faculty of Tropical Medicine, Mahidol University, Bangkok, Thailand; 2 Mahidol-Oxford Tropical Medicine Research Unit, Faculty of Tropical Medicine, Mahidol University, Bangkok, Thailand; 3 Department of Clinical Tropical Medicine, Faculty of Tropical Medicine, Mahidol University, Bangkok, Thailand; 4 Centre for Clinical Vaccinology and Tropical Medicine, Nuffield Department of Clinical Medicine, University of Oxford, Churchill Hospital, Oxford, United Kingdom; 5 Department of Military Medicine, Royal Centre for Defence Medicine, Birmingham, United Kingdom; 6 Department of Medicine, Faculty of Medicine, University of Kelaniya, Ragama, Sri Lanka; 7 Liverpool School of Tropical Medicine, Liverpool, United Kingdom; University Claude Bernard Lyon 1, France

## Abstract

**Background:**

Accuracy of rapid diagnostic tests for dengue infection has been repeatedly estimated by comparing those tests with reference assays. We hypothesized that those estimates might be inaccurate if the accuracy of the reference assays is not perfect. Here, we investigated this using statistical modeling.

**Methods/Principal Findings:**

Data from a cohort study of 549 patients suspected of dengue infection presenting at Colombo North Teaching Hospital, Ragama, Sri Lanka, that described the application of our reference assay (a combination of Dengue IgM antibody capture ELISA and IgG antibody capture ELISA) and of three rapid diagnostic tests (Panbio NS1 antigen, IgM antibody and IgG antibody rapid immunochromatographic cassette tests) were re-evaluated using Bayesian latent class models (LCMs). The estimated sensitivity and specificity of the reference assay were 62.0% and 99.6%, respectively. Prevalence of dengue infection (24.3%), and sensitivities and specificities of the Panbio NS1 (45.9% and 97.9%), IgM (54.5% and 95.5%) and IgG (62.1% and 84.5%) estimated by Bayesian LCMs were significantly different from those estimated by assuming that the reference assay was perfect. Sensitivity, specificity, PPV and NPV for a combination of NS1, IgM and IgG cassette tests on admission samples were 87.0%, 82.8%, 62.0% and 95.2%, respectively.

**Conclusions:**

Our reference assay is an imperfect gold standard. In our setting, the combination of NS1, IgM and IgG rapid diagnostic tests could be used on admission to rule out dengue infection with a high level of accuracy (NPV 95.2%). Further evaluation of rapid diagnostic tests for dengue infection should include the use of appropriate statistical models.

## Introduction

Dengue infection is a leading cause of illness and death in the tropics and subtropics. The causative organisms are mosquito-transmitted Dengue viruses, and patients may present with a range of clinical syndromes including viral syndrome, acute undifferentiated febrile illness, dengue fever, dengue hemorrhagic fever and dengue shock syndrome. On presentation, dengue infection often presents with symptoms and signs similar to other acute tropical infectious diseases, and a range of rapid diagnostic tests has been recommended for early diagnosis and patient management [1], [2].

There are two main methods for diagnosing dengue infection, namely virus and antibody detection. Virus detection includes viral isolation, polymerase chain reaction (PCR) and detection of nonstructural protein-1 (NS1) antigen. Antibody detection includes haemagglutination inhibition (HAI) tests and enzyme linked immunosorbent assay (ELISA) for detection of dengue IgM and IgG antibodies, usually using paired serum collections and assessing for a quantitative rise in antibody levels. Virus isolation and HAI are considered the gold standard techniques for virus and antibody detections, respectively, but are rarely used since they are time-consuming and laborious [3]. We have repeatedly used the Armed Forces Institute of Medical Sciences (AFRIMS) diagnostic serology methodologies on paired sera as a reference assay to determine the accuracy of alternative diagnostic tests [1], [4]–[7]. We hypothesized that the accuracy of this reference assay is imperfect, and that the accuracy of the alternative diagnostic tests estimated by comparing them with the reference assay might have been underestimated.

Bayesian latent class models (LCMs) have been increasingly used to evaluate the true accuracy of diagnostic tests in prospective cohort studies, as they do not require the assumption that any test is perfect [8]–[11]. The objective of this study was to use Bayesian LCMs to analyze existing data from a cohort of patients presenting to hospital with suspected dengue infection. We estimated the accuracy of three rapid diagnostic tests (Panbio NS1, IgM and IgG cassette tests), our reference assay for dengue infection, and the combination of all three rapid tests when used at clinical presentation.

## Materials and Methods

### Study patients and diagnostic tests

The data analyzed in this study was generated during a prospective cohort study of patients suspected of dengue infection. In brief, patients were recruited between June 2006 and June 2007 at Colombo North Teaching Hospital, Ragama, Sri Lanka. Inclusion criteria were the presence of fever (≥38°C) in patients aged 16 years or more who were suspected to have dengue infection. Blood samples were collected on admission and, where possible, at discharge and at follow-up 2–4 weeks later for convalescent-phase specimens. All specimens were stored at −85°C while at the clinical site and transported on dry ice to Bangkok, Thailand, for the test assessments. Reported elsewhere, a case-control study using samples from a subset of 259 of the patients recruited into the cohort was performed to evaluate six commercial point-of-care tests for acute dengue infections by comparing those tests with the reference assay [6].

**Figure 1:**
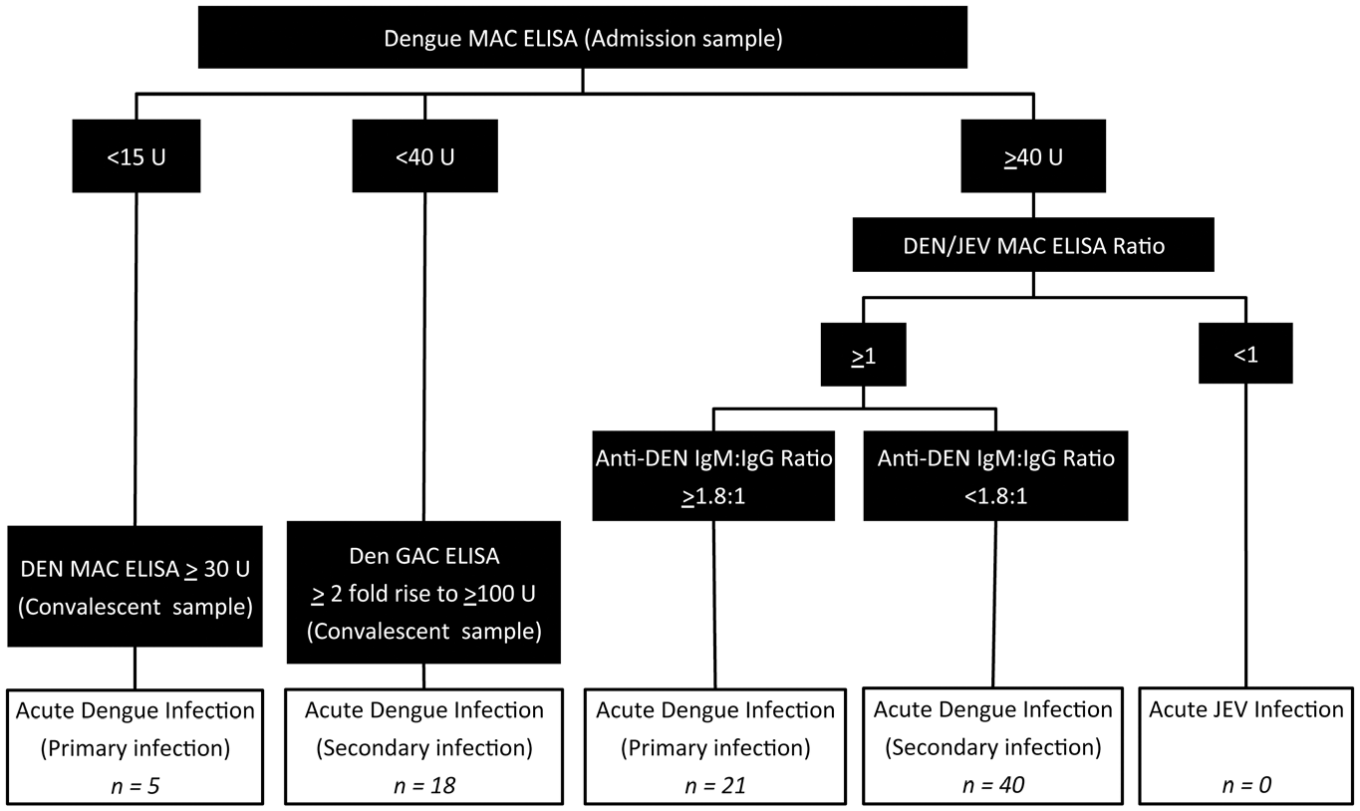
Flowchart showing the AFRIMS diagnostic algorithm for dengue infection.

For the purpose of the current study, all patients enrolled into the cohort were evaluated. In the cohort, every patient was tested with three rapid diagnostic tests including the Panbio first generation NS1 antigen strip, the Panbio Duo cassette IgM/IgG (Inverness, Australia), and our reference assay. NS1 antigen strip tests were performed on admission samples only. Dengue reference assays were performed at AFRIMS, Bangkok, Thailand as previously described (Figure 1) [7]. In short, AFRIMS tested paired admission and convalescent specimens using dengue (DEN) IgM antibody capture (MAC) ELISAs, IgG antibody capture (GAC) ELISA, and equivalent Japanese encephalitis virus (JEV) MAC and GAC ELISAs [12]–[16]. An acute primary dengue infection was diagnosed if (1) on the admission sample, the DEN MAC ELISA result was ≥40 units (U), the ratio of DEN MAC ELISA to JEV MAC ELISA results was ≥1 and the ratio of DEN MAC ELISA to DEN GAC ELISA results was ≥1.8∶1, or (2) the DEN MAC ELISA result on the admission sample was <15 U and in the convalescent-phase specimen ≥30 U. An acute secondary dengue infection was diagnosed if (1) on the admission sample, the DEN MAC ELISA result was ≥40 U, the ratio of DEN MAC ELISA to JEV MAC ELISA results was ≥1 and the ratio of DEN MAC ELISA to DEN GAC ELISA results was <1.8∶1, or (2) the DEN MAC ELISA on the admission sample was <40 U, the ratio of DEN GAC ELISA results on the convalescent-phase specimen compared to the admission specimen was ≥2 (2-fold rise) and the DEN GAC ELISA result on the convalescent-phase specimen was ≥100. An acute JEV infection was defined if the DEN MAC ELISA result on the admission sample was ≥40 U and the ratio of DEN MAC ELISA to JEV MAC ELISA results was <1.

### Ethics statement

Ethical approval for the cohort study was obtained from the Faculty of Medicine, University of Kelaniya in Sri Lanka, the Liverpool School of Tropical Medicine in the UK and the Walter Reed Army Institute of Research in the USA. Written informed consent was obtained from each subject enrolled into the study.

### Statistical analysis

#### AFRIMS serological assay as Gold Standard Model

Three rapid diagnostic test results (NS1 antigen strip, IgM cassette test and IgG cassette test) were analyzed using the reference assay results as the gold standard. Prevalence, sensitivities, specificities, positive and negative predictive values for the three rapid diagnostic tests were calculated with exact 95% confidence intervals using the Stata 11.0 statistical software package (Stata Corp., College Station, Texas).

#### Bayesian Latent Class Models

Use of LCMs and Bayesian LCMs to determine the accuracy of diagnostic tests when the accuracy of the gold standard is imperfect or unknown has been described in detail elsewhere [17], [18]. In brief, Bayesian LCMs do not assume that any test is perfect, but consider that each test could be imperfect in diagnosing the true disease status. The true disease status of the patient population is then defined on the basis of overall prevalence (the probability that a patient with suspected dengue is truly infected with dengue virus). LCMs estimate prevalence and accuracy of each test based on the observed frequency of the possible combinations of test results.

To estimate the accuracy of a diagnostic test by use of LCMs, the best-fitting model, as determined by the presence or absence of correlation between diagnostic tests in the model, should be used [19]. Possible correlations we evaluated were based on existing knowledge and external evidence. Therefore, correlations amongst serological tests (IgM, IgG and reference tests) were considered. The deviance information criterion (DIC) and Akaike information criterion (AIC) were used to evaluate goodness of model fit and to compare models. A difference in DIC or AIC of more than 10 indicated definite support to the model with the lower value, while a difference of between 5 and 10 was considered substantial, and less than 5 inconclusive. The best-fitted model was used to determine the accuracies of the three diagnostic tests and of their combinations on paired specimens. Then, the accuracies of the diagnostic tests on clinical presentation were determined by using test results for the admission samples only. All models assumed that no prior information (non-informative priors) about the unknown parameters (prevalence, sensitivities and specificities) was available. All parameters and associated 95% credible intervals (CrI) were estimated using WinBUGS 1.4 [20]. Text S1 and Text S2 provide full data sets and all of the models used, respectively.

#### Post-hoc model evaluation

The prediction accuracy of the final Bayesian LCM was evaluated by comparing the prevalence estimated and the final diagnoses made in the cohort study. Final diagnoses were based on microbiology results and physicians' judgment if all microbiology results were negative. Final diagnoses of dengue infection were categorized into four groups, following the dengue classification and case definitions of symptomatic dengue virus infections as described by WHO: (i) undifferentiated fever or viral syndrome, (ii) dengue fever, (iii) dengue hemorrhagic fever and (iv) dengue shock syndrome [21].

#### Sensitivity analysis

Sensitivity analyses were performed in which patients without convalescent samples were excluded and also in which different prior information were used [22], [23].

## Results

A total of 617 patients with fever and suspected dengue infection were included in the cohort study. Of these, 68 (11%) patients were excluded from further analysis because the result of at least one diagnostic test on the admission sample was not available. Of 549 patients included in the analysis, the median age was 35 years old (interquartile range [IQR], 25–50 years old), and 371 (68%) were male. Convalescent samples were available in 290 (52.8%) patients. Median time between onset of symptoms and collection of admission samples was 5 days (IQR, 3–8 days), and median time between onset of symptoms and collection of convalescent samples was 24 days (IQR, 19–30 days).

The final diagnoses of the 549 patients were dengue infection (135, 24.6%), chikungunya (102, 18.6%), leptospirosis (33, 6.0%), confirmed infections with no organism identified (38, 6.9%), various bacteraemias (23, 4.2%), confirmed non-infectious diseases causing fever (18, 3.2%), Q fever (17, 3.1%), rickettsial infections (14, 2.6%), tuberculosis (5, 0.9%), urinary tract infection (5, 0.9%), mumps (2, 0.4%), varicella (1, 0.2%), viral hepatitis (1, 0.2%), malaria (1, 0.2%), hantavirus infection (1, 0.2%) and undiagnosed febrile illnesses (184, 33.5%). The total number of diagnoses exceeds 549 as 31 cases had more than one diagnosis. Ten (1.8%) patients died in hospital due to leptospirosis (4, 40%), cerebrovascular accidents (3, 30%), liver failure (2, 20%) and leukaemia (1, 10%). Of 135 patients diagnosed with dengue infection, 131 had dengue fever, 4 had dengue hemorrhagic fever, and none had dengue shock syndrome.

Of 549 patients, 84 (15.3%) had positive results on the reference assay for dengue infection (26 primary infections and 58 secondary infections). None were positive for acute JEV infection. The NS1 antigen strip test, IgM cassette test and IgG cassette test were positive in 69, 91 and 147 patients, respectively. Only 13 patients (2.4%) were positive for the reference assay and all three rapid diagnostic tests.

### Reference assay as a perfect gold standard

We first assumed that the reference assay was a perfect gold standard (100% sensitivity and 100% specificity), and used this assumption to calculate sensitivities, specificities, PPVs and NPVs of the three rapid diagnostic tests and their combinations (Table 1). The estimated prevalence of dengue infection in the study population was 15.3%. The NS1 antigen test, IgM cassette test and IgG cassette test all had low sensitivity (54.8%, 50.0% and 61.9%, respectively) with variable specificity (95.1%, 89.5%, and 79.6%, respectively).

**Table 1:** Prevalence and sensitivities, specificities, and positive and negative predictive values (PPV's and NPV's) of diagnostic tests using the reference assay as gold standard and for final Bayesian latent class models.

Parameters	Reference assay as gold standard^a^	Final Bayesian latent class model^b^	Final Bayesian latent class model (using admission sample only)^b^
**Prevalence**	15.3 (12.4–18.6)	24.3 (19.1–30.0)	NA
**Reference assay**			
Sensitivity	100	62.0 (49.5–75.9)	NA
Specificity	100	99.6 (97.9–100)	NA
PPV	100	97.8 (89.7–99.9)	NA
NPV	100	89.1 (83.1–94.1)	NA
**Panbio NS1 antigen test**			
Sensitivity	54.8 (43.5–65.7)	45.9 (36.0–56.4)	NA
Specificity	95.1 (92.7–96.8)	97.9 (95.5–99.7)	NA
PPV	66.7 (54.3–77.6)	87.3 (74.0–98.1)	NA
NPV	92.1 (89.3–94.3)	84.9 (79.4–89.6)	NA
**Panbio Duo cassette IgM**			
Sensitivity	50.0 (38.9–61.1)	54.5 (45.4–63.8)	39.7 (35.2–44.1)
Specificity	89.5 (86.3–92.1)	95.5 (92.0–98.3)	96.6 (94.6–98.5)
PPV	46.2 (35.6–56.9)	79.5 (63.5–92.3)	79.2 (64.2–91.9)
NPV	90.8 (87.8–93.3)	86.8 (81.7–90.6)	83.3 (78.5–87.3)
**Panbio Duo cassette IgG**			
Sensitivity	61.9 (50.7–72.3)	62.1 (52.8–71.4)	42.6 (38.1–47.1)
Specificity	79.6 (75.6–83.1)	84.5 (80.1–88.5)	87.2 (85.4–89.1)
PPV	35.4 (27.7–43.7)	56.2 (44.6–67.6)	51.7 (41.3–61.6)
NPV	92.0 (88.9–94.5)	87.5 (82.2–91.6)	82.6 (77.5–86.8)
**Panbio NS1+IgM** c			
Sensitivity	79.8 (69.6–87.7)	78.9 (72.4–84.8)	72.8 (66.5–78.7)
Specificity	86.2 (82.8–89.2)	93.7 (90.7–96.8)	94.7 (91.9–97.5)
PPV	51.1 (42.3–60.0)	80.1 (68.2–90.6)	81.5 (69.8–91.6)
NPV	95.9 (93.6–97.6)	93.3 (89.5–95.9)	91.6 (87.5–94.5)
**Panbio NS1+IgM+IgG** c			
Sensitivity	92.9 (85.1–97.3)	91.7 (86.2–96.1)	87.0 (81.2–92.0)
Specificity	72.5 (68.2–76.5)	79.8 (76.6–83.3)	82.8 (79.9–86.0)
PPV	37.9 (31.2–44.9)	59.3 (48.9–69.0)	62.0 (51.8–71.4)
NPV	98.3 (96.2–99.4)	96.8 (93.9–98.7)	95.2 (91.9–97.5)

a Prevalence and accuracy of each test was estimated by the observed proportion classified by considering that the reference assay was perfect (100% sensitivity and 100% specificity). 95% confidence intervals (CI) were obtained in Stata 11.1.

b Prevalence and accuracy of each test was estimated by Bayesian latent class models by considering that the reference assay could be imperfect. Posterior estimates and 95% credible intervals (CrI) of each parameter were obtained in WinBUGs from 10,000 iterations of each of two chains starting from different initial values following a burn-in period of 5,000 iterations.

c A combination considers that positivity of either test is positive for dengue infection.

NA = Not applicable.

### Bayesian LCM

Bayesian LCMs were then applied to obtain an unbiased estimate of the accuracy of each diagnostic test. The models included all four diagnostic tests, including NS1 antigen test, IgM cassette test, IgG cassette test and our reference assay. First, we defined the best fitting Bayesian LCM by determining the presence of correlations between all three serological tests (IgM cassette test, IgG cassette test and our reference assay). Of the five plausible models (Table 2), the difference in DIC and AIC between the best fitting model (model 2) and the other four models were inconclusive (differences were less than 5). Table S1 shows the prevalence and accuracy of diagnostic tests estimated by all five models. In short, there was no substantial difference between all five models. Model 2, which had the lowest DIC and AIC, and included the correlation between IgM and IgG cassette tests, was selected as the best-fitted model.

**Table 2:** Description and model selection criteria.

Model	Correlation^a^	Scientific Background	DIC^b^	AIC^c^
0	None	It is possible that all (NS1, IgM, IgG and the reference assay) are not correlated.	113.9	110.3
1	IgM and the reference assay	IgM and the reference assay are based on IgM response. Both tests are more likely to be positive if the amount of IgM in blood in an infected subject is high, and to be negative if the amount of IgM in blood in the infected subject is low.	113.0	111.1
2	IgM and IgG	IgM and IgG are based on antibody response. Both tests are more likely to be positive if antibody response in an infected subject is high, and to be negative if antibody response in the infected subject is low.	108.5	106.7
3	IgG and the reference assay	IgG and the reference assay are based on IgG response. Both tests are more likely to be positive if the amount of IgG in blood in an infected subject is high, and to be negative if the amount of IgG in blood in the infected subject is low.	112.6	110.9
4	IgM, IgG and the reference assay	IgM, IgG and the reference assay are based on antibody response. All three tests are more likely to be positive if antibody response in an infected subject is high, and to be negative if antibody response in the infected subject is low.	NA	110.7

a All correlations were in infected subjects.

b **DIC** (deviance information criteria) is a generalization of AIC in a Bayesian setting. DIC was not applicable (NA) in model 4, which assumed a correlation between more than two tests.

c **AIC** (akaike information criteria) were used to evaluate goodness of model fit and to compare models.

A difference in DIC or AIC of more than 10 indicated definite support to the model with the lower value, while a difference of between 5 and 10 was considered substantial, and less than 5 inconclusive.

Using this model, the prevalence of dengue infection in the study population was estimated to be 24.3% (95% CrI 19.1%–30.0%,). The Bayesian LCM indicated that the reference assay had very high specificity (99.6, 95% CrI 97.9%–100%), but low sensitivity (62.0%, 95% CrI 49.5%–75.9%). Sensitivities and specificities of the Panbio NS1 (45.9% and 97.9%), IgM (54.5% and 95.5%) and IgG (62.1% and 84.5%) estimated by Bayesian LCM were significantly different from those estimated by assuming that the reference assay was perfect. Sensitivity and specificity for a combination of NS1 and IgM rapid tests, where a sample was defined as positive if either test was positive, were 78.9% and 93.7%, respectively. Sensitivity and specificity for a combination of NS1, IgM and IgG rapid tests, where a sample was defined as positive if any test was positive, were 91.7% and 79.8%, respectively (Table 1).

To determine the accuracy of the rapid serological tests on clinical presentation, only test results of IgM and IgG cassette test on admission samples were considered using the best-fitted Bayesian LCM. The sensitivity of IgM cassette test and IgG cassette test on clinical presentation was 39.7% and 42.6%, respectively. Sensitivity and specificity of a combination of NS1 and IgM were 72.8% and 94.7%, respectively, with positive predictive value (PPV) and negative predictive value (NPV) of 81.5% and 91.6%, respectively. Sensitivity and specificity of a combination of NS1, IgM and IgG rapid tests were 87.0% and 82.8%, respectively, with PPV and NPV of 62.0% and 95.2% respectively.

### Post-hoc model validation

According to the final diagnoses, 24.6% of the patients were classified as dengue infection (dengue fever or dengue hemorrhagic fever). This indicated that the estimated prevalence of dengue infection in the study population using Bayesian LCMs (24.3%) was credible.

### Sensitivity analysis

Sensitivity analysis was performed in which 259 of 549 (47.2%) patients without convalescent samples were excluded. By use of the best-fitted Bayesian LCM, the sensitivities of our reference assay, IgM cassette test and IgG cassette test were estimated to be 76.3% (95% CrI 59.2%–90.4%), 60.8% (95% CrI 49.5%–71.7%) and 68.3% (95% CrI 57.0%–78.3%), respectively, for patients suspected of dengue infection who had a convalescent sample. Specificity of those tests was not substantially different from the previous estimate, although all CrIs were wider as a consequence of the reduced sample sizes. There was no substantial change when different prior information was used (Table S1).

## Discussion

The key findings of this study are that the true sensitivity of our reference assay (AFRIMS MAC and GAC ELISA on paired serum) estimated by Bayesian LCM was very low (62.0%). The reduction in sensitivity of our reference assay from 100% assumed by the gold standard model to 62% as estimated by the Bayesian LCM model is due to the difference in the estimation methods. While the gold standard model assumed that our reference assay is perfect (sensitivity = 100%), Bayesian LCM estimated the true sensitivity of our reference assay using the results of every diagnostic test included in the model. Bayesian LCM also gave an estimated prevalence of 24.3% in patients who were suspected of dengue infection, compared with 15.3% based on our reference assay alone. This higher estimated prevalence is credible, since 24.6% patients had final diagnosis of dengue fever or dengue hemorrhagic fever based on the WHO definition and the exclusion of other diseases.

There are several potential explanations as to why our reference assay had such a low sensitivity in our setting. In common with other research and reflecting real life, we also failed to obtain a convalescent serum specimen from 47.2% of patients, either because they died, they refused to be bled on discharge, or they were lost to follow-up. The results from our sensitivity analysis show that sensitivity of our reference assay was 76.3% (95% CrI 59.2%–90.4%) in the ideal situation, in which convalescent samples were obtained from all patients. This increase in sensitivity is consistent with existing knowledge; however, this also suggests that a number of patients with dengue infection had a false-negative test result by our reference assay even if a convalescent-phase sample was available. Other possible explanations for the low sensitivity of our reference assay are that patients with dengue infection have variation in their immune response, insufficient time between paired serum collections, and that the cutoff level of DEN MAC and GAC ELISA used might not be optimal to detect some patients with true dengue infection [11].

Evaluation of diagnostic tests when the accuracy of the gold standard is unknown is an active area of biostatistical research, as the use of an imperfect gold standard to evaluate the accuracy of alternative tests is flawed and leads to biased results [18], [24]. Our study has shown that our reference assay represents a flawed reference standard against which to compare alternative diagnostic tests for dengue infection in a prospective study, and in this study we have demonstrated the usefulness of statistical models under such circumstances. For example, when compared with our reference assay, the IgM cassette test had a specificity of 89.5% (95% CI 86.3–92.1), representing a mediocre diagnostic test. When recalculated using Bayesian LCMs, the specificity of the IgM cassette test was 95.5% (95% CrI 92.0–98.3), representing a test with a high degree of specificity. The range of 95% CrI for specificity of the IgM cassette test estimated by the Bayesian LCM barely overlaps the range of 95% CI estimated by the gold standard model. When assessing the diagnostic utility of these rapid tests, use of estimates derived using Bayesian LCMs is preferable as they are unbiased by the false assumption that our reference assay is perfect.

Considering the true prevalence of dengue infection in a cohort population, Bayesian LCMs can be used to calculate unbiased estimates of PPV and NPV to determine the clinical usefulness of each diagnostic test and combinations of those tests. A combination of NS1, IgM and IgG on admission samples had an NPV of 95.2%, suggesting that negativity of all three tests could be used to rule out dengue infection with a high degree of accuracy in our setting. In addition, a combination of NS1 and IgM on the admission sample had a PPV of 81.5%, suggesting that positivity of either NS1 or IgM could be used to diagnose dengue infection with high level of confidence. This is consistent with many previous studies describing potential combinations of two or three tests in clinical setting [6], [16], [25]–[27]. It can be seen that the gold standard model minimally overestimates NPV for the combination of NS1, IgM and IgG performed on the admission samples compared to the Bayesian LCM (97.8 [95% CI 95.7–99.0] vs. 95.2 [95% CrI 91.9–97.5]), and markedly underestimates PPV for the combination of NS1 and IgM performed on the admission samples compared to the Bayesian LCM (47.1 [95% CI 37.8–56.4] vs. 81.5 [95% CrI 69.8–91.6]). Again, estimates by Bayesian LCM should be used because it does not falsely assume that our reference assay is perfect. Note that median duration of symptoms between onset of symptoms and collection of on-admission sample in our study was 5 days.

The data set inconclusively supported a positive correlation between the two serological tests detecting IgM and IgG immune response in patients with dengue, a finding that could be interpreted as meaning that both IgM and IgG cassette tests are more likely to be positive if the immune response is high, and to be negative if it is low. A positive correlation was not found between the IgM cassette test and the reference assay and between the IgG cassette test and the reference assay, even though our reference assays detect the response of both antibodies to dengue infection. Possible explanations are that the technology and antigens used for the IgM and IgG cassette tests were different from those used for the DEN MAC and GAC ELISA, respectively. This is supported by the simple tabulation and Kappa statistics that demonstrated poor agreement between the IgM cassette test and the reference assay (Kappa value = 0.38) and between the IgG cassette test and the reference assay (Kappa value = 0.32).

This study has several limitations. Using basic Bayesian LCMs to estimate the sensitivity and specificity of each test in a population does not allow us to determine the effect on these parameters of symptom duration, antimicrobials received prior to presentation, and timing of convalescent samples at the level of individual patients. These effects could be evaluated in advanced Bayesian LCMs [28]. Evaluation of other diagnostic tests, including viral isolation and HAI, was not done as those tests were not performed in our cohort study. PCR was performed in only a subset of patients who had our reference assay positive to determine serotype identity or who provided admission samples only [29]; therefore, PCR could not be assessed using Bayesian LCM in this study. It should be noted that the rapid diagnostic tests evaluated in this study are earlier versions of rapid tests for NS1, IgM and IgG. Currently available versions of these rapid diagnostic tests were only evaluated in the case-control data set of our cohort study; therefore, these newer tests could not be assessed using Bayesian LCM.

We conclude that our reference assay, a combination of AFRIMS DEN MAC and GAC ELISA on paired serum, has lower than expected sensitivity as it does not take dengue virus detection into consideration and hence is an imperfect gold standard against which to compare alternative diagnostic tests. Bayesian LCMs could be used to evaluate the accuracy of alternative diagnostic tests when the accuracy of the gold standard is unknown or is imperfect. On clinical presentation, a combination of the NS1, IgM and IgG cassette tests could be used as a set of rapid diagnostic tests for diagnosing dengue infection with a high level of accuracy.

## Supporting Information

Text S1 **Ragama fever study dataset.**
(DOC)

Text S2 **WinBUGS codes.**
(DOC)

Table S1 **Additional results.**
(DOC)
