# Supplementary material for: Estimating the True Accuracy of Diagnostic Tests for Dengue Infection Using Bayesian Latent Class Models
Source: PLoS One. 2013 Jan 18;8(1):e50765. doi: 10.1371/journal.pone.0050765 (PMC3548900; doi:10.1371/journal.pone.0050765)
Supplement: Text S1 — Ragama fever study dataset. (DOC) [file pone.0050765.s001.doc]

**Text S1** Ragama fever study dataset

The data used in Bayesian latent class models were structured as a matrix of test result profile of each individual subject (549 records). The value of 1 represents a positive and 0 represents a negative test result. Each column of matrix “y” represents Panbio NS1 antigen, Panbio IgM antibody, Panbio IgG antibody, reference assay, a combination between Panbio NS1 and IgM and a combination between Panbio NS1 IgM and IgG. Each row represents the result profile for each patient. Variable ‘sample.size’ represents the number of patients in the study, ‘no.test’ represents the the diagnostic tests being used in the likelihood (Panbio NS1 antigen, Panbio IgM antibody, Panbio IgG antibody and Reference assay), ‘permu’ represents all possible test result profiles. This is used to calculate the posterior predicted frequency of each profile.

# data

list(

sample.size=549,

no.test=4,

permu=16,

y=structure(.Data=c(

0,0,0,0,0,0,

0,0,0,0,0,0,

0,0,0,0,0,0,

1,1,1,1,1,1,

1,1,1,0,1,1,

1,0,0,0,1,1,

1,1,0,1,1,1,

0,0,0,0,0,0,

0,0,0,0,0,0,

0,0,0,0,0,0,

*(remaining data was omitted)*

0,0,0,0,0,0,

0,0,0,0,0,0,

0,0,0,0,0,0,

0,0,0,0,0,0,

0,0,0,0,0,0,

0,0,0,0,0,0,

1,1,0,1,1,1,

0,1,1,0,1,1,

0,1,0,0,1,1,

1,1,0,1,1,1),.Dim=c(549,6)) )
