# Supplementary material for: Estimating the True Accuracy of Diagnostic Tests for Dengue Infection Using Bayesian Latent Class Models
Source: PLoS One. 2013 Jan 18;8(1):e50765. doi: 10.1371/journal.pone.0050765 (PMC3548900; doi:10.1371/journal.pone.0050765)
Supplement: Text S2 — WinBUGS codes. (DOC) [file pone.0050765.s002.doc]

**Text S2 WinBUGS codes**

**Text S2.1** WinBUGS code for Model 0

# A Bernoullie model – conditional independence model (result of each diagnostic test on a given patient was independent conditional on true disease status of the patient)

#Model 0

#NS1: j = 1

#IgM: j = 2

#IgG: j = 3

#Reference assay: j = 4

#Combination of NS1 and IgM (either positive is positive): j = 5

#Combination of NS1, IgM and Ig G (any positive is positive): j = 6

#No random effect

model{

# Likelihood

for (i in 1:sample.size){

disease[i]~dbern(prev)

for (j in 1:no.test){

y[i,j]~dbern(p[i,j])

ypred[i,j]~dbern(p[i,j])

logit(p[i,j])<-disease[i]*alpha[j]+(1-disease[i])*beta[j]

accuracy[i,j]<-equals(disease[i],y[i,j])

}

}

# Combination

for (i in 1:sample.size){

for (j in 5:6){

positive.in.disease[i,j]<-disease[i]*y[i,j]

positive.in.nodisease[i,j]<-(1-disease[i])*y[i,j]

accuracy[i,j]<-equals(disease[i],y[i,j])

}

}

# Prior

prev~dbeta(1,1)

for (j in 1:no.test) {

s[j]~dbeta(1,1)

alpha[j]<-logit(s[j])

x[j]~dbeta(1,1)I(0.6,)

beta[j]<--logit(x[j])

}

# Prediction

for (i in 1:sample.size) {

for (k in 1:permu) {

for (j in 1:no.test) {

arraymatched.predicted[i,k,j]<-equals(ypred[i,j],profile[k,j])

arraymatched.observed[i,k,j]<-equals(y[i,j],profile[k,j])

}

nmatched.predicted[i,k]<-sum(arraymatched.predicted[i,k,])

matchedprofile.predicted[i,k]<-equals(nmatched.predicted[i,k],no.test)

nmatched.observed[i,k]<-sum(arraymatched.observed[i,k,])

matchedprofile.observed[i,k]<-equals(nmatched.observed[i,k],no.test)

}

}

for(k in 1:permu){

freq.predicted[k]<-sum(matchedprofile.predicted[,k])

freq.observed[k]<-sum(matchedprofile.observed[,k])

}

# Combination

for (j in 5:6){

s[j]<-sum(positive.in.disease[,j]) / sum(disease[])

x[j]<-1-sum(positive.in.nodisease[,j])/(sample.size-sum(disease[]))

}

# PPV and NPV

for (j in 1:6){

ppv[j]<- s[j]*prev / (s[j]*prev + (1-x[j])*(1-prev))

npv[j]<- x[j]*(1-prev) / (x[j]*(1-prev) + (1-s[j])*(prev))

acc[j]<- sum(accuracy[,j]) / sample.size

}

# Bayesian p value

for (k in 1:permu) {

pvalue[k]<-step(freq.predicted[k]-freq.observed[k])

}

}

**Text S2.2** WinBUGS code for Model 1 (Bernoulli model with random effect)

# Bernoulli model – conditional dependence between IgM and Reference assay (results of IgM and the reference assay of infected patients were correlated)

# Model 1 (Random effect)

# Random effect variable represented different IgM level in each infected subject

#Model 1

#NS1: j = 1

#IgM: j = 2

#IgG: j = 3

#Reference assay: j = 4

#Combination of NS1 and IgM (either positive is positive): j = 5

#Combination of NS1, IgM and Ig G (any positive is positive): j = 6

#Random effect, IgM, represents variation in IgM in diseased subject

model{

# Likelihood

for (i in 1:sample.size){

disease[i]~dbern(prev)

re[i] ~ dnorm(0,1)

for (j in 1:no.test){

y[i,j]~dbern(p[i,j])

ypred[i,j]~dbern(p[i,j])

accuracy[i,j]<-equals(disease[i],y[i,j])

}

#NS1

logit(p[i,1])<-disease[i]*alpha[1]+(1-disease[i])*beta[1]

#IgM

logit(p[i,2])<-disease[i]*alpha[2]+(1-disease[i])*beta[2]+disease[i]*IgM*re[i]

logit(positive.in.disease[i,2])<-disease[i]*alpha[2]+disease[i]*IgM*re[i]+(1-disease[i])*(-1000)

#IgG

logit(p[i,3])<-disease[i]*alpha[3]+(1-disease[i])*beta[3]

#Reference assay

logit(p[i,4])<-disease[i]*alpha[4]+(1-disease[i])*beta[4]+disease[i]*IgM*re[i]

logit(positive.in.disease[i,4])<-disease[i]*alpha[4]+disease[i]*IgM*re[i]+(1-disease[i])*(-1000)

}

# Combination

for (i in 1:sample.size){

for (j in 5:6){

positive.in.disease[i,j]<-disease[i]*y[i,j]

positive.in.nodisease[i,j]<-(1-disease[i])*y[i,j]

accuracy[i,j]<-equals(disease[i],y[i,j])

}

}

# Prior

prev~dbeta(1,1)

IgM~dnorm(0,0.1)I(0,)

for (j in 1:no.test) {

s[j]~dbeta(1,1)

alpha[j]<-logit(s[j])

x[j]~dbeta(1,1)I(0.4,)

beta[j]<--logit(x[j])

}

se[1] <- s[1]

se[2] <- sum(positive.in.disease[,2])/sum(disease[])

se[3] <- s[3]

se[4] <- sum(positive.in.disease[,4])/sum(disease[])

# Prediction (similar to Text S2.1)

# Predicted and observed frequency (similar to Text S2.1)

# Combination

for (j in 5:6){

s[j]<-sum(positive.in.disease[,j]) / sum(disease[])

x[j]<-1-sum(positive.in.nodisease[,j])/(sample.size-sum(disease[]))

se[j] <- s[j]

}

# PPV and NPV

for (j in 1:6){

ppv[j]<- se[j]*prev / (se[j]*prev + (1-x[j])*(1-prev))

npv[j]<- x[j]*(1-prev) / (x[j]*(1-prev) + (1-se[j])*(prev))

acc[j]<- sum(accuracy[,j]) / sample.size

}

# Bayesian p value (similar to Text S2.1)

}

**Text S2.3** WinBUGS code for Model 2 (Bernoulli model with random effect)

# Bernoulli model – conditional dependence between IgM and IgG (results of IgM and IgG of infected patients were correlated)

# Model 2 (Random effect)

# Random effect variable represented different IgM and IgG antibodies level in each infected subject

#Model 2

#NS1: j = 1

#IgM: j = 2

#IgG: j = 3

#Reference assay: j = 4

#Combination of NS1 and IgM (either positive is positive): j = 5

#Combination of NS1, IgM and Ig G (any positive is positive): j = 6

#Random effect, IgMG, represents variation in IgM and IgG in diseased subject

model{

# Likelihood

for (i in 1:sample.size){

disease[i]~dbern(prev)

re[i] ~ dnorm(0,1)

for (j in 1:no.test){

y[i,j]~dbern(p[i,j])

ypred[i,j]~dbern(p[i,j])

accuracy[i,j]<-equals(disease[i],y[i,j])

}

#NS1

logit(p[i,1])<-disease[i]*alpha[1]+(1-disease[i])*beta[1]

#IgM

logit(p[i,2])<-disease[i]*alpha[2]+(1-disease[i])*beta[2]+disease[i]*IgMG*re[i]

logit(positive.in.disease[i,2])<-disease[i]*alpha[2]+disease[i]*IgMG*re[i]+(1-disease[i])*(-1000)

#IgG

logit(p[i,3])<-disease[i]*alpha[3]+(1-disease[i])*beta[3]+disease[i]*IgMG*re[i]

logit(positive.in.disease[i,3])<-disease[i]*alpha[3]+disease[i]*IgMG*re[i]+(1-disease[i])*(-1000)

#Reference assay

logit(p[i,4])<-disease[i]*alpha[4]+(1-disease[i])*beta[4]

}

# Combination

for (i in 1:sample.size){

for (j in 5:6){

positive.in.disease[i,j]<-disease[i]*y[i,j]

positive.in.nodisease[i,j]<-(1-disease[i])*y[i,j]

accuracy[i,j]<-equals(disease[i],y[i,j])

}

}

# Prior

prev~dbeta(1,1)

IgMG~dnorm(0,0.1)I(0,)

for (j in 1:no.test) {

s[j]~dbeta(1,1)

alpha[j]<-logit(s[j])

x[j]~dbeta(1,1)I(0.4,)

beta[j]<--logit(x[j])

}

se[1] <- s[1]

se[2] <- sum(positive.in.disease[,2])/sum(disease[])

se[3] <- sum(positive.in.disease[,3])/sum(disease[])

se[4] <- s[4]

# Prediction (similar to Text S2.1)

# Predicted and observed frequency (similar to Text S2.1)

# Sensitivity and specificity of the combinations (similar to Text S2.2)

# PPV and NPV (similar to Text S2.2)

# Bayesian p value (similar to Text S2.1)

}

**Text S2.4** WinBUGS code for Model 3 (Bernoulli model with random effect)

# Bernoulli model – conditional dependence between IgG and the reference assay (results of IgG and the reference assay of infected patients were correlated)

# Model 3 (Random effect)

# Random effect variable represented different IgG level in each infected subject

#Model 3

#Bernoulli model - conditional dependence, re, between IgG and the reference assay

#NS1: j = 1

#IgM: j = 2

#IgG: j = 3

#Reference assay: j = 4

#Combination of NS1 and IgM (either positive is positive): j = 5

#Combination of NS1, IgM and Ig G (any positive is positive): j = 6

#Random effect, IgG, represents variation in IgG in diseased subject

model{

# Likelihood

for (i in 1:sample.size){

disease[i]~dbern(prev)

re[i] ~ dnorm(0,1)

for (j in 1:no.test){

y[i,j]~dbern(p[i,j])

ypred[i,j]~dbern(p[i,j])

accuracy[i,j]<-equals(disease[i],y[i,j])

}

#NS1

logit(p[i,1])<-disease[i]*alpha[1]+(1-disease[i])*beta[1]

#IgM

logit(p[i,2])<-disease[i]*alpha[2]+(1-disease[i])*beta[2]

#IgG

logit(p[i,3])<-disease[i]*alpha[3]+(1-disease[i])*beta[3]+disease[i]*IgG*re[i]

logit(positive.in.disease[i,3])<-disease[i]*alpha[3]+disease[i]*IgG*re[i]+(1-disease[i])*(-1000)

#Reference assay

logit(p[i,4])<-disease[i]*alpha[4]+(1-disease[i])*beta[4]+disease[i]*IgG*re[i]

logit(positive.in.disease[i,4])<-disease[i]*alpha[4]+disease[i]*IgG*re[i]+(1-disease[i])*(-1000)

}

# Combination

for (i in 1:sample.size){

for (j in 5:6){

positive.in.disease[i,j]<-disease[i]*y[i,j]

positive.in.nodisease[i,j]<-(1-disease[i])*y[i,j]

accuracy[i,j]<-equals(disease[i],y[i,j])

}

}

# Prior

prev~dbeta(1,1)

IgG~dnorm(0,0.1)I(0,)

for (j in 1:no.test) {

s[j]~dbeta(1,1)

alpha[j]<-logit(s[j])

x[j]~dbeta(1,1)I(0.4,)

beta[j]<--logit(x[j])

}

se[1] <- s[1]

se[2] <- s[2]

se[3] <- sum(positive.in.disease[,3])/sum(disease[])

se[4] <- sum(positive.in.disease[,4])/sum(disease[])

# Prediction (similar to Text S2.1)

# Predicted and observed frequency (similar to Text S2.1)

# Sensitivity and specificity of the combinations (similar to Text S2.2)

# PPV and NPV (similar to Text S2.2)

# Bayesian p value (similar to Text S2.1)

}

**Text S2.5** WinBUGS code for Model 4 (Bernoulli model with random effect)

# Bernoulli model – conditional dependence between all serological tests (results of IgM, IgG and the Reference assay of infected patients were correlated)

# Model 4 (Random effect)

# Random effect variable represented different antibodies level in each infected subject

#Model 4

#NS1: j = 1

#IgM: j = 2

#IgG: j = 3

#Reference assay: j = 4

#Combination of NS1 and IgM (either positive is positive): j = 5

#Combination of NS1, IgM and Ig G (any positive is positive): j = 6

#Random effect, Ab, represents variation in antibodies level (IgM, IgG and reference assay) in diseased subject

model{

# Likelihood

for (i in 1:sample.size){

disease[i]~dbern(prev)

re[i] ~ dnorm(0,1)

#NS1

y[i,1]~dbern(p[i,1])

ypred[i,1]~dbern(p[i,1])

logit(p[i,1])<-disease[i]*alpha[1]+(1-disease[i])*beta[1]

accuracy[i,1]<-equals(disease[i],y[i,1])

for (j in 2:no.test){

y[i,j]~dbern(p[i,j])

ypred[i,j]~dbern(p[i,j])

logit(p[i,j])<-disease[i]*alpha[j]+(1-disease[i])*beta[j]+disease[i]*Ab*re[i]

logit(positive.in.disease[i,j])<-disease[i]*alpha[j]+disease[i]*Ab*re[i]+(1-disease[i])*(-1000)

accuracy[i,j]<-equals(disease[i],y[i,j])

}

}

# Combination

for (i in 1:sample.size){

for (j in 5:6){

positive.in.disease[i,j]<-disease[i]*y[i,j]

positive.in.nodisease[i,j]<-(1-disease[i])*y[i,j]

accuracy[i,j]<-equals(disease[i],y[i,j])

}

}

# Prior

prev~dbeta(1,1)

Ab~dnorm(0,0.1)I(0,)

for (j in 1:no.test) {

s[j]~dbeta(1,1)

alpha[j]<-logit(s[j])

x[j]~dbeta(1,1)I(0.4,)

beta[j]<--logit(x[j])

}

se[1] <- s[1]

for (j in 2:no.test){

se[j] <-sum(positive.in.disease[,j])/sum(disease[])

}

# Prediction (similar to Text S2.1)

# Predicted and observed frequency (similar to Text S2.1)

# Sensitivity and specificity of the combinations (similar to Text S2.2)

# PPV and NPV (similar to Text S2.2)

# Bayesian p value (similar to Text S2.1)

}
