# Supplementary material for: Estimating the True Accuracy of Diagnostic Tests for Dengue Infection Using Bayesian Latent Class Models
Source: PLoS One. 2013 Jan 18;8(1):e50765. doi: 10.1371/journal.pone.0050765 (PMC3548900; doi:10.1371/journal.pone.0050765)
Supplement: Table S1 — Additional results. (DOC) [file pone.0050765.s003.doc]

**Table S1 additional results**

**Table S1.1 Prevalence, sensitivities and specificities (95% credible interval) for different diagnostic tests of dengue using Bayesian latent class and random-effects models in full data set (N=549)**

| **Parameters** | **Model 0^a^** | **Model 1^b^** | **Model 2^c^** | **Model 3^d^** | **Model 4^e^** |
| --- | --- | --- | --- | --- | --- |
| **Prevalence** | 23.8 (18.4 – 29.8) | 23.9 (18.3 – 30.2) | 24.3 (19.1 – 30.0) | 24.1 (18.4 – 30.3) | 24.6 (18.7 – 31.6) |
| **Reference assay** |  |  |  |  |  |
| Sensitivity | 63.0 (50.1 – 77.9) | 62.5 (49.4 – 77.6) | 62.0 (49.5 – 75.9) | 62.1 (49.1 – 77.4) | 60.9 (47.5 – 76.9) |
| Specificity | 99.4 (97.7 – 100) | 99.4 (97.7 – 100) | 99.6 (97.9 – 100) | 99.4 (97.7 – 100) | 99.5 (97.7 – 100) |
| PPV | 97.3 (88.5 – 99.9) | 97.3 (88.7 – 99.9) | 97.8 (89.7 – 99.9) | 97.2 (88.8 – 100) | 97.4 (88.8 – 99.9) |
| NPV | 89.6 (83.4 – 94.9) | 89.5 (82.7 – 94.9) | 89.1 (83.1 – 94.1) | 89.2 (82.6 – 94.8) | 88.7 (81.2 – 94.5) |
| **Panbio NS1 antigen test** |  |  |  |  |  |
| Sensitivity | 45.4 (35.3 – 56.9) | 45.5 (35.2 – 57.3) | 45.9 (36.0 – 56.4) | 45.3 (35.3 – 57.4) | 45.6 (35.1 – 57.8) |
| Specificity | 97.5 (95.3 – 99.3) | 97.6 (95.2 – 99.3) | 97.9 (95.5 – 99.7) | 97.6 (95.4 – 99.5) | 98.0 (95.6 – 99.8) |
| PPV | 84.7 (72.0 – 95.6) | 85.4 (72.2 – 96.0) | 87.3 (74.0 – 98.1) | 85.8 (72.6 – 97.0) | 88.2 (73.9 – 99.1) |
| NPV | 85.2 (79.3 – 90.3) | 85.1 (79.1 – 90.4) | 84.9 (79.4 – 89.6) | 85.0 (78.8 – 90.4) | 84.7 (77.8 – 90.2) |
| **Panbio Duo cassette IgM** |  |  |  |  |  |
| Sensitivity | 55.9 (45.9 – 66.1) | 55.1 (45.3 – 65.2) | 54.5 (45.4 – 63.8) | 55.5 (45.3 – 65.3) | 53.4 (42.7 – 64.2) |
| Specificity | 95.6 (91.8 – 98.7) | 95.5 (91.6 – 98.6) | 95.5 (92.0 – 98.3) | 95.7 (91.6 – 99.0) | 95.4 (91.3 – 98.6) |
| PPV | 79.7 (61.8 – 94.1) | 79.3 (60.9 – 93.9) | 79.5 (63.5 – 92.3) | 80.3 (60.9 – 95.5) | 79.1 (60.7 – 94.1) |
| NPV | 87.5 (82.4 – 91.4) | 87.2 (82.0 – 91.3) | 86.8 (81.7 – 90.6) | 87.2 (82.0 – 91.3) | 86.4 (80.1 – 90.9) |
| **Panbio Duo cassette IgG** |  |  |  |  |  |
| Sensitivity | 63.7 (54.0 – 73.4) | 63.6 (53.8 – 72.9) | 62.1 (52.8 – 71.4) | 62.6 (52.6 – 72.2) | 61.6 (50.7 – 71.9) |
| Specificity | 84.7 (80.2 – 88.7) | 84.7 (80.2 – 89.1) | 84.5 (80.1 – 88.5) | 84.5 (80.0 – 88.7) | 84.5 (80.0 – 88.8) |
| PPV | 56.5 (43.7 – 68.4) | 56.8 (43.3 – 69.7) | 56.2 (44.6 – 67.6) | 56.2 (42.9 – 68.3) | 56.4 (43.0 – 69.1) |
| NPV | 88.3 (83.2 – 92.3) | 88.2 (82.9 – 92.2) | 87.5 (82.2 – 91.6) | 87.8 (82.2 – 92.0) | 87.1 (80.6 – 91.8) |
| **Panbio NS1 + IgM ^f^** |  |  |  |  |  |
| Sensitivity | 79.6 (72.9 – 85.4) | 79.0 (71.8 – 85.1) | 78.9 (72.4 – 84.8) | 79.4 (72.6 – 85.3) | 78.2 (69.6 – 84.8) |
| Specificity | 93.4 (90.1 – 96.8) | 93.4 (90.0 – 96.7) | 93.7 (90.7 – 96.8) | 93.7 (90.0 – 97.4) | 93.7 (90.2 – 97.4) |
| PPV | 79.1 (66.0 – 90.4) | 79.0 (65.6 – 90.5) | 80.1 (68.2 – 90.6) | 80.0 (65.9 – 92.4) | 80.2 (66.6 – 92.4) |
| NPV | 93.7 (89.8 – 96.2) | 93.4 (89.2 – 96.2) | 93.3 (89.5 – 95.9) | 93.5 (90.0 – 96.1) | 93.0 (87.9 – 96.0) |
| **Panbio NS1 + IgM + IgG ^f^** |  |  |  |  |  |
| Sensitivity | 93.2 (88.8 – 97.2) | 93.0 (88.2 – 97.0) | 91.7 (86.2 – 96.1) | 92.7 (87.8 – 96.9) | 91.8 (84.2 – 96.6) |
| Specificity | 79.8 (76.1 – 83.8) | 79.9 (76.0 – 84.1) | 79.8 (76.6 – 83.3) | 79.9 (76.0 – 84.1) | 80.0 (76.2 – 84.4) |
| PPV | 59.0 (47.8 – 69.7) | 59.3 (47.4 – 70.5) | 59.3 (48.9 – 69.0) | 59.4 (47.7 – 70.7) | 59.9 (48.0 – 71.8) |
| NPV | 97.4 (95.1 – 99.1) | 97.4 (94.8 – 99.0) | 96.8 (93.9 – 98.7) | 97.2 (94.5 – 99.0) | 96.8 (92.5 – 98.8) |

^a^ The model assumes that NS1, IgM, IgG and the reference assay are not correlated.

^b^ The model assumes that IgM and the reference assay are correlated based on IgM response i.e. both tests are more likely to be positive if the amount of IgM in blood in an infected subject is high, and to be negative if the amount of IgM in blood in the infected subject is low.

^c^ The model assumes that IgM and IgG tests are correlated based on antibody response i.e. both tests are more likely to be positive if antibody response in an infected subject is high, and to be negative if antibody response in the infected subject is low.

^d^ The model assumes that IgG and the reference assay are correlated based on IgG response i.e. both tests are more likely to be positive if the amount of IgG in blood in an infected subject is high, and to be negative if the amount of IgG in blood in the infected subject is low.

^e^ IgM, IgG and the reference assay are correlated based on antibody response i.e. all three tests are more likely to be positive if antibody response in an infected subject is high, and to be negative if antibody response in the infected subject is low.

^f^A combination considers that positivity of either test is positive for dengue infection.

**Table S1.2** Prevalence, sensitivities and specificities for different diagnostic tests of dengue using Bayesian latent class and random-effects models in full data set (N=549) and in a subset of patients who have convalescent samples (N=290)

| **Parameters** | **Final Bayesian model in**  **full data set**  **(95% credible interval)** | **Final Bayesian model in**  **patients with convalescent samples**  **(95% credible interval)** |
| --- | --- | --- |
| **Prevalence** | 24.3 (19.1 – 30.0) | 25.9 (19.8 – 33.7) |
| **Reference assay** |  |  |
| Sensitivity | 62.0 (49.5 – 75.9) | 76.3 (59.2 – 90.4) |
| Specificity | 99.6 (97.9 – 100) | 98.9 (95.9 – 100) |
| PPV | 97.8 (89.7 – 99.9) | 96.1 (85.1 – 99.8) |
| NPV | 89.1 (83.1 – 94.1) | 92.3 (84.0 – 97.3) |
| **Panbio NS1 antigen test** |  |  |
| Sensitivity | 45.9 (36.0 – 56.4) | 58.2 (43.6 – 72.8) |
| Specificity | 97.9 (95.5 – 99.7) | 99.5 (97.5 – 100) |
| PPV | 87.3 (74.0 – 98.1) | 97.7 (88.3 – 100) |
| NPV | 84.9 (79.4 – 89.6) | 87.3 (78.9 – 92.9) |
| **Panbio Duo cassette IgM** |  |  |
| Sensitivity | 54.5 (45.4 – 63.8) | 60.8 (49.5 – 71.7) |
| Specificity | 95.5 (92.0 – 98.3) | 89.0 (82.9 – 94.8) |
| PPV | 79.5 (63.5 – 92.3) | 65.7 (48.5 – 84.6) |
| NPV | 86.8 (81.7 – 90.6) | 86.7 (80.9 – 91.2) |
| **Panbio Duo cassette IgG** |  |  |
| Sensitivity | 62.1 (52.8 – 71.4) | 68.3 (57.0 – 78.3) |
| Specificity | 84.5 (80.1 – 88.5) | 75.4 (68.7 – 82.1) |
| PPV | 56.2 (44.6 – 67.6) | 49.2 (36.3 – 64.1) |
| NPV | 87.5 (82.2 – 91.6) | 87.2 (81.1 – 91.9) |
| **Panbio NS1 + IgM ^a^** |  |  |
| Sensitivity | 78.9 (72.4 – 84.8) | 87.7 (81.3 – 94.0) |
| Specificity | 93.7 (90.7 – 96.8) | 89.0 (85.7 – 94.1) |
| PPV | 80.1 (68.2 – 90.6) | 73.7 (62.2 – 87.2) |
| NPV | 93.3 (89.5 – 95.9) | 95.4 (91.4 – 98.1) |
| **Panbio NS1 + IgM + IgG ^a^** |  |  |
| Sensitivity | 91.7 (86.2 – 96.1) | 94.5 (89.9 – 98.7) |
| Specificity | 79.8 (76.6 – 83.3) | 70.5 (67.5 – 75.7) |
| PPV | 59.3 (48.9 – 69.0) | 52.9 (42.7 – 65.3) |
| NPV | 96.8 (93.9 – 98.7) | 97.4 (94.3 – 99.5) |

^a^A combination considers that positivity of either test is positive for dengue infection.

**Table S1.3** Prevalence, sensitivities and specificities for different diagnostic tests of dengue using Bayesian latent class and random-effects models with non-informative, sceptical and enthusiastic priors

| Parameters | Bayesian model with non-informative priors  (95% credible interval) ^a^ | Bayesian model with sceptical priors  (95% credible interval) ^b^ | Bayesian model with enthusiastic priors  (95% credible interval) ^c^ |
| --- | --- | --- | --- |
| Prevalence | 24.3 (19.1 – 30.0) | 23.2 (18.0 – 28.6) | 24.2 (18.9 – 30.0) |
| Reference assay |  |  |  |
| Sensitivity | 62.0 (49.5 – 75.9) | 63.8 (51.6 – 77.3) | 63.7 (51.3 – 77.4) |
| Specificity | 99.6 (97.9 – 100) | 99.0 (97.1 – 99.8) | 99.8 (98.4 – 100) |
| PPV | 97.8 (89.7 – 99.9) | 95.0 (85.7 – 99.2) | 99.1 (92.3 – 100) |
| NPV | 89.1 (83.1 – 94.1) | 90.1 (84.6 – 94.8) | 89.6 (83.7 – 94.6) |
| Panbio NS1 antigen test |  |  |  |
| Sensitivity | 45.9 (36.0 – 56.4) | 46.9 (36.6 – 58.1) | 47.5 (37.8 – 57.9) |
| Specificity | 97.9 (95.5 – 99.7) | 97.3 (95.0 – 99.1) | 98.1 (95.8 – 99.9) |
| PPV | 87.3 (74.0 – 98.1) | 84.1 (70.4 – 94.9) | 88.6 (75.5 – 99.7) |
| NPV | 84.9 (79.4 – 89.6) | 85.9 (80.1 – 90.5) | 85.4 (80.1 – 90.1) |
| Panbio Duo cassette IgM |  |  |  |
| Sensitivity | 54.5 (45.4 – 63.8) | 55.2 (46.1 – 64.8) | 55.6 (46.5 – 64.7) |
| Specificity | 95.5 (92.0 – 98.3) | 94.8 (91.2 – 97.6) | 95.5 (92.0 – 98.4) |
| PPV | 79.5 (63.5 – 92.3) | 76.1 (60.0 – 89.3) | 80.0 (64.1 – 92.9) |
| NPV | 86.8 (81.7 – 90.6) | 87.6 (83.0 – 91.4) | 87.1 (82.4 – 91.0) |
| Panbio Duo cassette IgG |  |  |  |
| Sensitivity | 62.1 (52.8 – 71.4) | 62.9 (53.9 – 72.0) | 63.0 (53.8 – 71.9) |
| Specificity | 84.5 (80.1 – 88.5) | 84.0 (79.6 – 87.9) | 84.6 (80.3 – 88.4) |
| PPV | 56.2 (44.6 – 67.6) | 54.2 (42.3 – 65.4) | 56.5 (45.3 – 67.1) |
| NPV | 87.5 (82.2 – 91.6) | 88.3 (83.5 – 92.2) | 87.8 (82.6 – 91.8) |
| Panbio NS1 + IgM ^d^ |  |  |  |
| Sensitivity | 78.9 (72.4 – 84.8) | 80.0 (73.4 – 85.9) | 79.4 (73.4 – 84.5) |
| Specificity | 93.7 (90.7 – 96.8) | 93.0 (90.0 – 95.8) | 93.8 (90.6 – 97.0) |
| PPV | 80.1 (68.2 – 90.6) | 77.5 (65.3 – 87.4) | 80.2 (67.8 – 91.3) |
| NPV | 93.3 (89.5 – 95.9) | 93.9 (90.5 – 96.4) | 93.5 (90.0 – 95.8) |
| Panbio NS1 + IgM + IgG ^d^ |  |  |  |
| Sensitivity | 91.7 (86.2 – 96.1) | 92.5 (87.3 – 96.9) | 91.9 (87.2 – 95.8) |
| Specificity | 79.8 (76.6 – 83.3) | 79.0 (75.9 – 82.2) | 79.7 (76.5 – 83.3) |
| PPV | 59.3 (48.9 – 69.0) | 57.1 (46.8 – 66.4) | 59.0 (48.8 – 69.0) |
| NPV | 96.8 (93.9 – 98.7) | 97.3 (94.6 – 99.0) | 96.9 (94.3 – 98.5) |

^a^ Beta(1,1) was selected as non-informative priors and used for sensitivities and specificities of every test. This prior assumed that no prior information (non-informative priors) about the unknown parameters (prevalence, sensitivities and specificities) was available.

^b^ Beta(2,2) was selected as sceptical priors and used for sensitivities and specificities of every test. This prior strongly believed that sensitivity and specificity of each test is around 50%, and there is less than 2% chance that sensitivity and specificity of each test is more than 95% or less than 5%.

^c^ Beta(3.5,0.5) was selected as enthusiastic priors and used for sensitivities and specificities of every test. This prior strongly believed that sensitivity and specificity of each test is close to 93%, and there is less than 5% chance that sensitivity and specificity of each test is than 50%.

^d^ A combination considers that positivity of either test is positive for dengue infection.
